# Neurosurgical resection of multiple brain metastases: outcomes, complications, and survival rates in a retrospective analysis

**DOI:** 10.1007/s11060-024-04744-w

**Published:** 2024-06-21

**Authors:** Sebastian Niedermeyer, M. Schmutzer-Sondergeld, J. Weller, S. Katzendobler, S. Kirchleitner, R. Forbrig, P. N. Harter, L. V. Baumgarten, C. Schichor, V. Stoecklein, N. Thon

**Affiliations:** 1 Department of Neurosurgery, LMU Hospital, Ludwig-Maximilian-University Munich, Marchioninistrasse 15, 81377 Munich, Germany; 2 Department of Neuroradiology, LMU Hospital, Ludwig-Maximilian-University Munich, Marchioninistrasse 15, 81377 Munich, Germany; 3 Center for Neuropathology and Prion Research, LMU Hospital, Ludwig-Maximilian- University Munich, Feodor-Lynen Strasse 23, 81377 Munich, Germany; 4 German Cancer Consortium (DKTK), Partner Site Munich, and German Cancer Research Center (DKFZ), Heidelberg, Germany

**Keywords:** Multiple brain metastases, Surgical resection, Survival rates, Complications

## Abstract

**Purpose:**

This study investigates the outcomes of microsurgical resection of multiple brain metastasis (BMs).

**Methods:**

This retrospective, monocentric analysis included clinical data from all consecutive BM patients, who underwent simultaneous resection of ≥ 2 BMs between January 2018 and May 2023. Postoperative neurological and functional outcomes, along with perioperative complications, as well as survival data were evaluated.

**Results:**

A total of 47 patients, with a median age of 61 years (IQR 48–69), underwent 73 craniotomies (median 2; range 1–3) for resection of 104 BMs. Among patients, 80.8% presented with symptomatic BMs, causing focal neurological deficits in 53% of cases. Gross total resection was achieved in 87.2% of BMs. Karnofsky Performance Scale (KPS) scores improved in 42.6% of patients, remained unchanged in 46.8%, and worsened in 10.6% after surgery. Perioperative complications were observed in 29.8% of cases, with transient complications occurring in 19.2% and permanent deficits in 10.6%. The 30-days mortality rate was 2.1%. Logistic regression identified eloquent localization (*p* = 0.036) and infratentorial craniotomy (*p* = 0.018) as significant predictors of postoperative complications. Concerning overall prognosis, patients with permanent neurological deficits post-surgery (HR 11.34, *p* = 0.007) or progressive extracranial disease (HR: 4.649; *p* = 0.006) exhibited inferior survival.

**Conclusion:**

Microsurgical resection of multiple BMs leads to clinical stabilization or functional improvement in most patients. Although transient complications do not affect overall survival, the presence of persistent neurological deficits (> 3 months post-surgery) and progressive extracranial disease negatively impact overall survival. This highlights the importance of careful patient selection for resection of multiple BMs.

**Supplementary Information:**

The online version contains supplementary material available at 10.1007/s11060-024-04744-w.

## Introduction

The progress in prevention, screening and treatment has extended the lifespan of cancer patients, subsequently leading to an increased incidence of brain metastasis (BMs) [1–4]. Epidemiological studies suggest that up to 40% of cancer patients will eventually develop BMs, with nearly half of them presenting with multiple lesions [5, 6]. Historical data reported generally poor prognosis for BMs with a median survival ranging from 3 to 6 months following whole-brain radiation therapy (WBRT) [7–9]. Prognostic factors for patients undergoing treatment of BMs include age, Karnofsky Performance scale (KPS) score and extracranial tumor status [10–12]. These prognostic factors have been validated for surgery of single BMs, helping to improve patients selection [13].

Generally, microsurgery remains the cornerstone treatment for larger lesions (> 3 cm) and those causing significant mass effect (> 1 cm midline shift) [14, 15]. While current Class I evidence supports the resection of a single BM followed by radiotherapy, particularly in patients exhibiting a good performance status, the simultaneous microsurgical resection of multiple BMs in one procedure has been performed in selected cases [14, 16–18]. However, only a few studies have explored the risks and benefits of this approach [16–19]. The identification of crucial prognostic factors guiding the selection of patients for simultaneous resection of multiple BMs remains an unanswered query. Notably, postoperative deficits are associated with decreased survival following single BM resection [20] similar to what is seen in glioblastoma patients [21, 22]. With this study we assess the risks and benefits of simultaneous resection of multiple BMs, aiming to improve selection criteria for patients suitable for microsurgery of multiple BMs.

## Methods

### Study design, setting and participants

This retrospective analysis included all consecutive patients, who underwent simultaneous resection of multiple BMs at our institution between January 2018 and May 2023. Patients with multiple BMs were typically referred to our interdisciplinary neuro-oncology board by their primary oncologists. According to our internal guidelines, we considered surgical resection for lesions that were causing acute neurological symptoms due to their location and/or were too large for radiosurgical treatment. As part of the joint management decision, patients were scheduled for tumor bed irradiation after resection and, where applicable, stereotactic irradiation for additional lesions. Thus, our study also included 20 patients who had multiple BMs but underwent surgical resection of only a limited number of them. This study received approval from the independent ethics committee of our medical center (reference no. 23–0223) and adhered to institutional guidelines.

### Preoperative variables

Clinical notes, imaging studies, and operative notes of eligible patients were reviewed for demographic data, primary tumor diagnosis, previous treatment and preoperative functional status, as assessed by KPS. Clinical assessment encompassed a spectrum of focal deficits, such as motor deficits, sensory deficits, aphasia, visual deficits, and cerebellar symptoms. We also considered the presence and frequency of seizures and signs indicative of elevated intracranial pressure.

Extracranial disease status was classified as follows: (1) stable—primary tumor site with/-out extracranial metastases reported as stable in imaging studies within the 3 months preceding surgery; (2) progressive—evidence of primary tumor and/or extracranial disease progression within the 3 months prior to BM resection; (3) synchronous— primary tumor discovered as a result of BM as the first manifestation of the disease.

Patients were further stratified based on the Radiation Therapy Oncology Group (RTOG) recursive partitioning analysis derived prognostic classes [10, 23].

Imaging studies, conducted by radiologists/ neuroradiologists, included defining BMs number, size and location on preoperative magnetic resonance imaging (MRI) as well as assessment of extent of resection as being defined by early (< 72 h) postoperative MRI controls.

Tumor location was classified according to Sawaya et al. [24] as follows: (1) eloquent regions encompassing the primary motor or sensory cortex, calcarine fissure, expressive or receptive speech cortex, dentate nucleus or brainstem; (2) areas near eloquent regions; (3) non-eloquent regions.

Volumetric analysis of BM was conducted using T1-weighted contrast-enhanced MRI, employing the smart brush tool (*Brainlab AG, Munich, Germany*). For volumetric assessment of post-operative residual tumor T1-weighted and postcontrast T1- weighted images were overlaid to differentiate the blood products from enhancing residual tumor.

### Surgical indication and technique

Microsurgical resection was recommended for multiple symptomatic BMs or when the size or mass effects precluded focal irradiation protocols. Salvage surgery addressed post-radiation growing and/or symptomatic BMs, suspicious of a recurrence. All procedures were conducted under total intravenous anesthesia. To minimize operation time, we avoided changing the patient`s position whenever possible, with position changes made in only three patients. In one patient, however, this strategy may have increased the risk of perioperative thromboembolic complications in the semi-sitting position. The choice of skin incision was contingent on the specifics of the craniotomy, typically involving one or more linear incisions. Frameless navigation (*Brainlab AG, Munich, Germany*) was used to determine the size and number of craniotomies needed to ensure a minimal-invasive and safe resection of multiple BMs. In instances involving two distinct lesions, the preference was for two separate craniotomies over a single larger craniotomy. This approach aimed to mitigate the perioperative risk e.g. of postoperative epidural hematoma formation. In case of eloquent localization of the targeted BMs with critical involvement or proximity to the senso-motor cortex and/ or the pyramidal tract, intraoperative neuromonitoring with continuous transcranial and direct cortical stimulation was applied. According to our in house standard operating procedures, all patients with multiple BM resections were routinely monitored in intensive care postoperatively.

### Outcome assessment

Symptomatic improvement was defined as an improvement in specific neurological deficits or KPS. Symptomatic worsening indicated new neurological deficits and/ or worsened KPS. Deficits that lasted up to 3 months or longer than 3 months were classified as “transient” versus “persistent”, respectively. Perioperative death was defined as any death occurring within 30 days after surgery. Complications were categorized based on Sawaya et al. (1998) into three groups [24]: (1) neurological complications, directly resulting in neurological deficits; (2) regional complications, related to the surgical site but not causing neurological deficits; and (3) systemic complications, involving medical issues distant from the surgical site.

Each patient underwent an MRI within 72 h post-surgery. Gross total resection (GTR) was achieved when no residual tumor was visible on postoperative T1-weighted (with/-out contrast enhancement) or T2-weighted MR sequences, while patients with tumor remnants were classified as having undergone subtotal resection.

### Statistical methods

Descriptive statistics were presented as mean ± standard deviation or as median ± interquartile range (IQR). Non-Gaussian distribution were confirmed using the Shapiro-Wilk tests. The Wilcoxon matched-pairs signed rank test assessed KPS differences and tumor volumes before and after treatment. Postoperative overall survival (OS) was analyzed using the Kaplan-Meier method. Logistic regression identified predictors of postoperative symptomatic improvement, and Cox proportional hazard analysis assessed the association of variables with OS. For all statistical analysis a *p*-value < 0.05 was deemed to be significant. All statistical tests were performed with GraphPad Prism 10 (GraphPad Software, La Jolla, California).

## Results

### Descriptive data of patients and surgeries

Our study comprised 47 patients with a median age of 61 years (IQR 48–69). Patient characteristics are summarized in Table 1. The median time from the initial diagnosis of tumor disease to surgery for BMs was 24 months, with a maximum duration of 124 months from the first diagnosis. Notably, in 23.4% of cases (*n* = 11), the diagnosis of BMs preceded the initial diagnosis of tumor disease. When classified by RPA, 17% of patients belonged to RPA class 1, 70.2% to RPA class 2, 12.8% to RPA class 3.

**Table 1:** Patient’s characteristics

variable	
no. of patients	47
age (years); median [IQR]	61 [48; 69]
primary tumor entity; n (%)	
NSCLC	16 (34)
malignant melanoma	11 (23.4)
breast cancer	7 (14.9)
colorectal cancer	4 (8.5)
ovarian cancer	3 (6.4)
gastric cancer	2 (4.3)
others	4 (8.5)
extracranial disease	
stable	30 (63.8)
progressive	6 (12.8)
synchronous	11 (23.4)
preoperative KPS ≥ 70	39 (83)
preoperative KPS < 70	8 (17)
presenting symptoms	
asymptomatic	9 (19.2)
motor/sensory deficit	10 (21.3)
aphasia/dysarthira	6 (12.8)
hemianopsia / visual deficit	4 (8.5)
headache	5 (10.6)
signs of elevated ICP	6 (12.8)
seizures	7 (14.9)
RPA classification; n (%)	
class 1	8 (17)
class 2	33 (70.2)
class 3	6 (12.8)
time from first diagnosis; median [IQR]	24 [1; 64]

A total of 104 metastasis were surgically removed, and their characteristics are delineated in Table 2. The majority of patients (80.9%) underwent simultaneous resection of two BMs, while the remaining (19.1%) underwent simultaneous resection of more than two BMs.

Nearly half of the patients underwent a single (46.8%), the remaining underwent two or more craniotomies (52.2%). Among all craniotomies, 44.7% involved both hemispheres. Regarding postoperative care, the median length of stay in the ICU was 1 day (range 1–20 days), with a median hospital stay of 7 days (range 3–27 days).

**Table 2:** Surgical characteristics

variable	
total number of BMs resected	104
number of metastasis resected in one surgery	
2; n (%)	38 (80.9)
≥ 2; n (%)	9 (19.1)
location of resected BM	
eloquent	33 (31.7)
near eloquent	20 (19.2)
non eloquent	51 (49)
number of craniotomies; n (%)	73
1	22 (46.8)
≥ 2	25 (53.2)
location of craniotomies; n (%)	
supratentorial	59 (80.8)
infratentorial	14 (19.2)
leftsided craniotomy; n (%)	13 (22)
rightsided craniotomy; n (%)	18 (30.5)
craniotomy on both sides; n (%)	28 (47.5)
upfront surgery; n (%)	38 (80.9)
salvage surgery; n (%)	9 (19.1)
time form previous radiation; median [IQR]	7 [3; 24]
intraoperative neuromonitoring; n (%)	11 (23.4)
intraoperative blood loss (ml); median [IQR]	250 [150; 400]
length of operation (hours); median [IQR]	3 [2; 4]
length of ICU stay (days); median (range)	1 (1–20)
length of hospital stay (days); median (range)	7 (3–27)

### Surgical resection and functional outcome

The median preoperative intracranial volume, encompassing all BMs, among patients undergoing resection, was 16.2 cm^3^. Moreover, 20 patients had additional BMs that were not scheduled for resection with a median volume of 3.8 cm³ (IOR 2–11). Following resection, MRI scans revealed GTR in 87.2% of patients (*n* = 41), with residual tumor in 12.8% (*n* = 6). Notably, there was a significant reduction in overall intracranial tumor burden postoperatively (determined by Wilcoxon matched pairs signed rank test, *p* < 0.0001).

After surgery, an immediate clinical improvement was observed in 46.8% of patients, with 42.6% showing no significant change, and 10.6% experiencing a worsening with respect to the KPS score. Upon 3-month follow-up 52.2% demonstrated improvement compared to their preoperative status, while 32.6% remained unchanged, and 15.2% experienced a decline.

During the last follow-up (median 6 months, IQR 3–12), the status of patients varied, with 21.2% still showing improvement, 27.3% remaining unchanged, but also 51.5% experiencing a worsening of their condition. The Wilcoxon matched-pairs signed-rank test was employed to assess individual changes in KPS, exhibiting a significant improvement (*p* = 0.0004) at discharge.

At 3 months-follow up symptomatic worsening (total *n* = 6) was attributed to cerebral complications/new permanent deficits in 5 out of 6 patients, while in 1 out of 6 patients symptomatic worsening was attributed to decrease of general condition. At last follow-up, among 17 patients with symptomatic worsening, 3 experienced deteriorations due to cerebral progression, 3 patients due to cerebral and systemic progression and 11 due to systemic progression alone.

Examining the documented (disease-related) causes of death in our hospital revealed that 2 patients succumbed to cerebral progression, while 7 patients succumbed to systemic disease progression.

### Surgical complications and mortality

The study identified complications in 29.8% of patients, with 9 individuals (19.2%) requiring revision surgery (Table 3). Transient neurological complications were registered in 12.7% and resolved before discharge.

**Table 3:** Surgical complications including neurological, regional and systemic complications

variable	transient; *n*(%)	persistent; *n*(%)
**neurological complications**		
motor or sensory deficit	2 (4.3)	2 (4.3)
apahsia/dysphasia	2 (4.3)	2 (2.1)
visual field deifict	0	0
vigilance disorder	2 (4.3)	1 (2.1)
**regional complications**		
surgical site infection; n (%)	1 (2.1)	0
CSF fistula; n (%)	1 (2.1)	0
**systemic complications**		
pulmonary embolism; n (%)	1 (2.1)	0

Notably, one patient (2.1% of the entire cohort) experienced surgery-related death due to spontaneous bleeding in a smaller infratentorial metastasis after resection of a supratentorial metastasis. Among the variables potentially associated with postoperative complications infratentorial craniotomy (OR 5.444; *p* = 0.018) and eloquent tumor location (OR 4.267; *p* = 0.036) emerged as statistically significant. Demographic factors like age (*p* = 0.201), preoperative KPS score (*p* = 0.242), but also surgical factors like number of BMs resected (*p* = 0.219 or extent of resection (*p* = 0.527), did not influence the occurrence of postoperative complications (see Supplementary Information).

### Postoperative treatment

Within the cohort, 35 patients underwent SRS to the tumor bed. Two patients who had undergone salvage surgery and had prior SRS received WBRT for multiple lesions. Nine patients had previously undergone radiotherapy and did not require re-radiation. Notably, cerebral recurrence (*n* = 6) predominantly occurred distantly from the site of BMs resection in 66.7%, while 33.3% were characterized as local recurrences. Adjuvant therapy included SRS and chemotherapy in 18 patients. Four patients who had prior radiotherapy underwent adjuvant chemotherapy alone. Importantly, surgery enabled adjuvant chemotherapy in five out of six patients with progressive extracranial disease and nine out of 11 patients with synchronous extracranial disease.

### Overall survival after resection of multiple metastasis

**Fig. 1:**
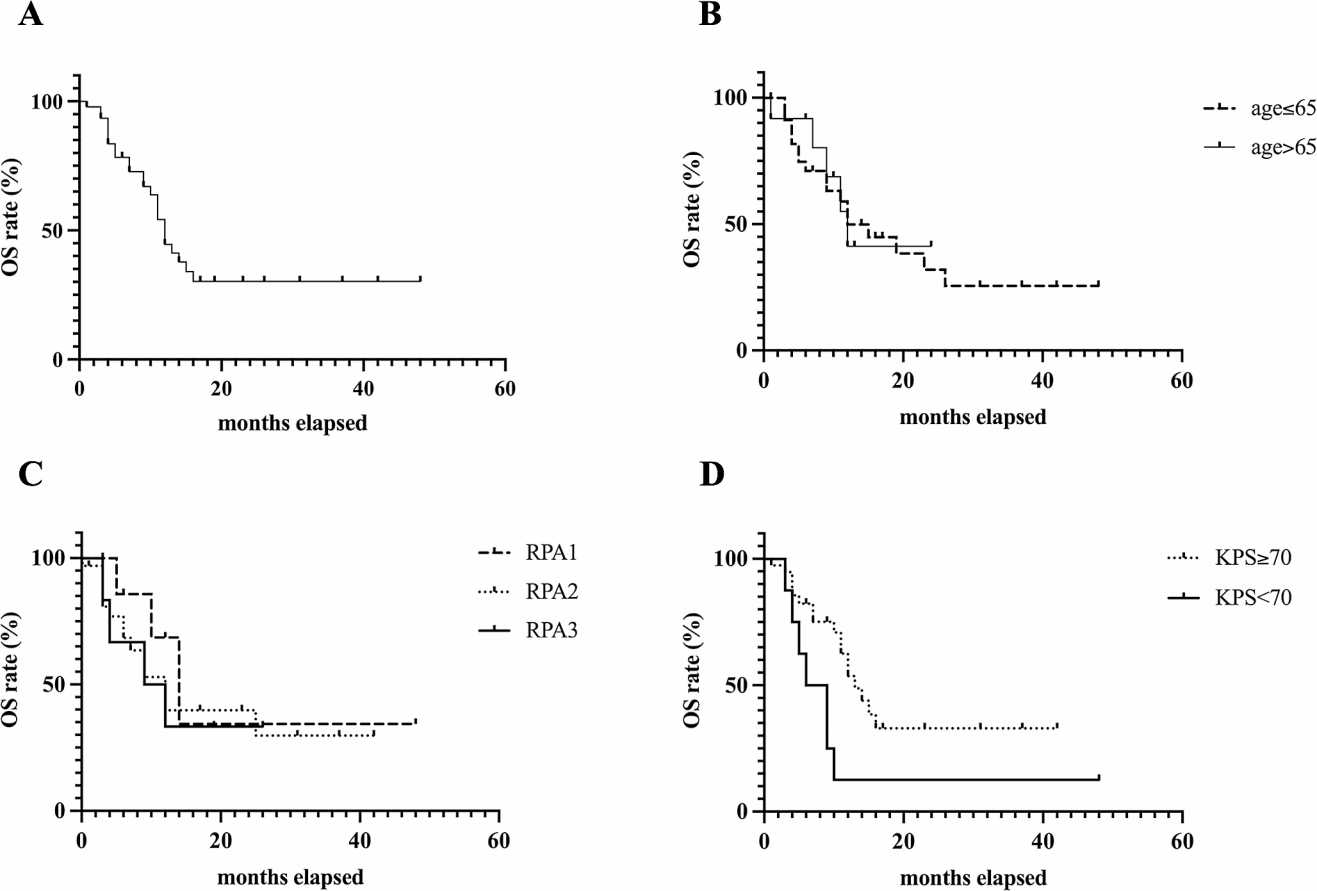
Graphs showing survival curves for patients with multiple BMs undergoing surgery (**A**) OS of all patients (**B**) OS of patients separated by age (**C**) OS of patients separated by RPA classes (**D**) overall survival of patients separated by KPS at admission

Median OS following resection of multiple BMs was 12 months. The log-rank test demonstrated no significant differences in OS between patients under 65 years and those aged 65 years and older (log-rank test, *p* = 0.917). Similarly, although median survival decreased from 14 months, to 12 months, to 10.5 months in RPA classes 1,2 and 3, respectively, these differences did not reach statistical significance (*p* = 0.635). Significantly longer OS was observed in patients with preoperative KPS score ≥ 70 (median 13 months), compared to those with a KPS score < 70 (median OS 7.5months; log-rank test, *p* = 0.024) (Fig. 1). Importantly, patients with a KPS score < 70 after resection of multiple BMs demonstrated a median OS of only 4 months. OS was analyzed for the four most frequent primary tumors revealing the following trend: Patients with multiple BMs from breast cancer had the longest OS following resection, with a median of 16 months. Median survival decreased for NSCLC, malignant melanoma and colorectal cancer, with medians of 12 months, 11.5 months and 8 months, respectively (log rank test, *p* = 0.678).

Cox regression analysis revealed that progressive extracranial disease and permanent postoperative deficits after surgery significantly affected OS in both univariate (HR 4.828; *p* = 0.002 and HR 11.96; *p* = 0.002, respectively) and multivariate analyses (HR 4.649; *p* = 0.006 and HR 11.34; *p* = 0.007, respectively) (Table 4). Neither age (*p* = 0.484) nor tumor characteristics such as tumor volume (*p* = 0.633) influenced OS. Previous radiation influenced OS significantly in univariate (HR 3.772; *p* = 0.016) but not multivariate regression. Additionally, subtotal resection demonstrated no impact on OS (*p* = 0.249).

**Table 4:** Cox regression analysis to evaluate the influence of perioperative variables on survival. Variables with a significance level of *p* < 0.05 in univariate regression were included in the multivariate regression analysis

Variable			univariate						multivariate				
			Hazard ratio		95%CI		*p*		Hazard ratio		95%CI		*p*
age	< 65		0.727		0.309–1.902		0.484						
	≥ 65		1.376		0.526–3.242								
preoperative KPS score	≥ 70		0.863		0.360–2.387		0.756						
	< 70		1.159		0.419–2.775								
postoperative deficits	transient		0.615		0.203–1.537		0.336						
	persistent		11.96		2.249–57.74		0.002**		11.34		1.823–68.95		0.007**
status of extracranial disease	stable		0.445		0.187–1.001		0.055						
	progressive		4.828		1.661–12.62		0.002**		4.649		1.473–13.22		0.006**
	synchronous		1.385		0.560–3.158		0.454						
preoperative tumor volume			0.992		0.955–1.018		0.633						
previous radiation	yes		3.772		1.167–10.69		0.016*		1.595		0.400-5.446		0.478
	no		0.265		0.094–0.857								
extent of resection	GTR		0.308		0.017–1.470		0.249						
	STR		3.249		0.680–58.23								

## Discussion

### Resection of multiple BMs improves functional status in the majority of patients

Previous studies, focusing on single BMs have demonstrated an improved functional status following resection [25]. In our cohort, the majority of patients (80.8%) underwent surgery primarily due to symptomatic lesions. Aligning with these findings, we registered an overall improvement in functional status in patients with multiple BMs (*p* = 0.0004 ) upon discharge. The median hospital stay was 7 days, slightly longer than previously reported for craniotomy procedures with a median of 4 to 5 days [24, 26]. This prolonged duration could be attributed to factors such as the advanced median age of 61 years in our cohort and postoperative complications, both of which are known to impact hospital stay length [26].

### Infratentorial craniotomy and eloquent location are significantly associated with postoperative complications

In our cohort, the overall complication rate was 29.8%, with a mortality rate of 2.1%, aligning with complications rates following resection of multiple BMs reported by other research groups ranging from 11.8 to 50%, and mortality rates from 1.9 to 11.1%, respectively [16–19]. Comparatively, the complication rates reported for the resection of single metastasis seem to be slightly lower, ranging from 9.1 to 23.5% [18, 27]. It’s essential to note that our classification of perioperative complications encompasses transient deteriorations expected solely due to surgery in eloquent location, without ischemia, etc., with subsequent rapid clinical improvement. Within our cohort, eloquent localization (*p* = 0.036) and infratentorial craniotomy (*p* = 0.018) were significantly associated with postoperative complications.

Despite the transient nature of most complications, these results underscore the importance of careful patient selection and surgical planning to optimize outcomes and minimize risks [28]. A study conducted by Paek et al., which included 76 patients with multiple BMs, of whom 17 patients underwent resection of multiple BMs suggested treatment strategies for multiple BMs based on RPA classification [18].

### Functional status, not RPA class or age is associated with overall survival post-resection of multiple BMs

Despite the prognostic significance of RPA classification in studies involving single brain metastasis resection, our analysis did not reveal significant differences in OS between RPA classes (*p* = 0.635) following resection and adjuvant therapy for BM [13]. It has been noted that the RPA classification may overly emphasize age as a predictive factor [29–32]. Examining the OS of patients aged 65 years and older compared to those younger than 65 years following resection of multiple BM, we found no significant difference (*p* = 0.917). This reinforces previous research suggesting that frailty as a state of increased vulnerability resulting from different age-related conditions may hold greater predictive value than chronological age alone [32, 33].

Functional status has emerged as a pivotal predictor of OS in single BMs [34]. Consistent with these observations, we found that patients undergoing surgical resection of multiple BMs with a preoperative KPS ≥ 70 exhibited a statistically significant longer OS compared to those with a preoperative KPS score < 70 (*p* = 0.024). In summary, our analysis underscores the importance of functional assessment in guiding the selection of patients for multiple BMs resection. Moreover, a rigid adherence to age-related cut-off values, which also influence Recursive Partitioning Analysis classification [35], should be avoided.

### Permanent deficits post-surgery and progressive systemic disease negatively impact the OS of patients

Extent of resection has shown to be associated with OS in patients with glioblastoma, but its impact on BMs is debated [34, 36, 37].

In our cohort, we achieved GTR rate of 87.2%, falling within the reported range of 67–91% for single BM resection, depending on eloquence of the tumor site [18]. Importantly, the presence of residual tumor did not adversely affect OS (*p* = 0.249). This may be attributed to modern treatment modalities such as SRS, which provide good local control (even in case of incomplete resections) [37]. Accordingly, all patients (without prior radiotherapy) also routinely received postoperative tumor bed irradiation in our series.

Progressive extracranial disease significantly impacted OS (*p* = 0.006), emphasizing the importance of vigilance in managing systemic disease in patients with multiple BMs. Accordingly, the primary cause of mortality in the cohort was systemic disease progression, accounting for 77.8% of cases. This is in line with previous studies reporting 72% of death due to systemic disease progression [18]. Importantly, surgery led to stabilization or improvement of functional status in most patients, thereby allowing 82.4% of patients with progressive or synchronous extracranial disease access to chemotherapy.

In 23.4% of our patients, BM represented the initial manifestation of the tumor disease, and often, assessment of extracranial disease occurred after surgery. Cox regression analysis revealed that synchronous extracranial disease had no adverse impact on OS (*p* = 0.454). With the emergence of novel treatment modalities for various cancer types, the presence of multiple BMs or synchronous extracranial metastases may not necessarily contraindicate surgery [38–40].

Previous research focusing on surgery for single BMs as well as glioblastoma patients has emphasized the detrimental effect of new postoperative deficits on OS [20–22]. Permanent deficits following surgery also adversely impacted OS in our study (*p* = 0.007), with patients exhibiting a postoperative KPS < 70 demonstrating a median OS of only 4 months. Hence, while the benefits of enhanced functionality outweigh transient deterioration, it is imperative to address and mitigate the risk of permanent deficits, as they markedly worsen overall prognosis. In situations where concerns regarding permanent deficits arise, alternative treatments such as stereotactic radiosurgery (SRS), including fractionated approaches if necessary, may offer improved outcomes if feasible [41, 42].

Finally, analyzing cerebral recurrence patterns revealed predominantly distant site recurrences (66.7%), with local recurrences accounting for 33.3%. This observation may be attributed to the impact of SRS, which, while having fewer deleterious effects compared to whole-brain radiation therapy (WBRT), exhibits lower rates of distant control [2, 43, 44].

### Limitations

This study is subject to the inherent limitations of retrospective research. While it represents a substantial series of multiple BMs resections, the relatively low patient numbers restrict the ability to draw generalizable conclusions and limit statistical power. Additionally, we were unable to include a control group of patients with multiple symptomatic and/or large BMs treated with radiosurgery or systemic therapy. Instead, the study primarily aims to demonstrate the feasibility of multiple resections in a single surgery and facilitate informed risk-benefit discussions between treating physicians and patients.

## Conclusion

In conclusion, our study underscores the positive impact of resection in improving functional status for most patients with multiple BMs and enabling access to chemotherapy for systemic disease. Our data indicate that age is not associated with survival following local treatment. However, a worsened clinical condition must be critically considered when indicating resection for multiple lesions, as patients with a KPS < 70 show significantly reduced overall survival. Regarding periprocedural risk assessment, tumors located in eloquent and/or infratentorial regions are associated with a higher rate of postoperative complications, which, although usually transient, may delay further treatment. When combined with local radiotherapeutic strategies (tumor bed irradiation plus radiosurgery for additional BMs), effective intracranial tumor control can be achieved, meeting the prerequisites for systemic tumor therapy. It remains unclear to what extent new tumor drugs with central nervous system efficacy can supplement or even replace these local therapy concepts.

### Electronic supplementary material

Below is the link to the electronic supplementary material.

Supplementary Material 1

## Data Availability

The datasets generated during and/or analysed during the current study are available from the corresponding author on reasonable request.
