# Supplementary material for: Neurosurgical resection of multiple brain metastases: outcomes, complications, and survival rates in a retrospective analysis
Source: J Neurooncol. 2024 Jun 21;169(2):349–58. doi: 10.1007/s11060-024-04744-w (PMC11341644; doi:10.1007/s11060-024-04744-w)
Supplement: Supplementary file 1 — Supplementary Material 1 [file 11060_2024_4744_MOESM1_ESM.docx]

**Supplementary Information** Univariate logistic regression was performed to analyze the impact of variables on operative morbidity. Due to a limited number of events, multivariate logistic regression was not feasible.

| **Variable** |  | **odds ratio of postoperative complication** |  | **95%CI** |  | ***p*** |
| --- | --- | --- | --- | --- | --- | --- |
|  |  |  |  |  |  |  |
| **age** | **≤65** | 0.42 |  | 0.109 - 1.612 |  | 0.201 |
|  | **>65** | 2.381 |  | 0.620 - 9.204 |  |  |
| **preoperative KPS score** | **≥70** | 3.692 |  | 0.579 - 72.45 |  | 0.242 |
|  | **<70** | 0.271 |  | 0.014 - 1.728 |  |  |
| **previous radiation** | **yes** | 0.271 |  | 0.014 - 1.728 |  | 0.242 |
|  | **no** | 3.692 |  | 0.579 -72.45 |  |  |
| **craniotomy location** | **supratentorial** | 0.184 |  | 0.043 - 0.729 |  | **0.018*** |
|  | **infratentorial** | 5.444 |  | 1.371 - 23.51 |  |  |
| **tumor location** | **eloquent** | 4.267 |  | 1.133 - 17.67 |  | **0.036*** |
|  | **near-eloquent** | 1.667 |  | 0.459 - 6.236 |  | 0.437 |
|  | **noneloquent** | 0.75 |  | 0.191 to 2.743 |  | 0.667 |
| **no. of BMs resected** | **2** | 0.388 |  | 0.084 - 1.851 |  | 0.219 |
|  | **>2** | 2.578 |  | 0.540 - 11.93 |  |  |
| **primary tumor** | **lung** | 0.259 |  | 0.037 - 1.165 |  | 0.110 |
|  | **melanoma** | 0.485 |  | 0,067 - 2.294 |  | 0.401 |
|  | **breast** | 2.25 |  | 0.388 - 12.01 |  | 0.338 |
| **extent of resection** | **GTR** | 2.069 |  | 0.291 - 41.83 |  | 0.527 |
|  | **STR** | 0.483 |  | 0.024 - 3.434 |  |  |
